# Ethanol-induced cerebellar transcriptomic changes in a postnatal model of fetal alcohol spectrum disorders: Focus on disease onset

**DOI:** 10.3389/fnins.2023.1154637

**Published:** 2023-03-16

**Authors:** Kalee N. Holloway, James C. Douglas, Tonya M. Rafferty, Ania K. Majewska, Cynthia J. M. Kane, Paul D. Drew

**Affiliations:** ^1^Department of Neurobiology and Developmental Sciences, University of Arkansas for Medical Sciences, Little Rock, AR, United States; ^2^Department of Neuroscience, University of Rochester Medical Center, Rochester, NY, United States; ^3^Department of Neurology, University of Arkansas for Medical Sciences, Little Rock, AR, United States

**Keywords:** FASD, microglia, astrocytes, oligodendrocytes, transcriptomics

## Abstract

Fetal alcohol spectrum disorders (FASD) are a group of neurodevelopmental disorders caused by ethanol exposure *in utero*, which can result in neurocognitive and behavioral impairments, growth defects, and craniofacial anomalies. FASD affects up to 1-5% of school-aged children in the United States, and there is currently no cure. The underlying mechanisms involved in ethanol teratogenesis remain elusive and need greater understanding to develop and implement effective therapies. Using a third trimester human equivalent postnatal mouse model of FASD, we evaluate the transcriptomic changes induced by ethanol exposure in the cerebellum on P5 and P6, after only 1 or 2 days of ethanol exposure, with the goal of shedding light on the transcriptomic changes induced early during the onset and development of FASD. We have highlighted key pathways and cellular functions altered by ethanol exposure, which include pathways related to immune function and cytokine signaling as well as the cell cycle. Additionally, we found that ethanol exposure resulted in an increase in transcripts associated with a neurodegenerative microglia phenotype, and acute- and pan-injury reactive astrocyte phenotypes. Mixed effects on oligodendrocyte lineage cell associated transcripts and cell cycle associated transcripts were observed. These studies help to elucidate the underlying mechanisms that may be involved with the onset of FASD and provide further insights that may aid in identifying novel targets for interventions and therapeutics.

## Introduction

Fetal Alcohol Spectrum Disorders (FASD) are a leading cause of preventable developmental abnormalities around the world, and result in a range of clinical outcomes that can include craniofacial anomalies, neurological malformations, cognitive and behavioral impairment, and growth defects (Hoyme et al., 2016). FASD are not only devastating to affected individuals, but also have significant economic impact globally (Greenmyer et al., 2018; Sokol, 2018). The global prevalence of FASD varies by region, with a mean of 0.8 percent (Lange et al., 2017). The prevalence has been estimated to be as high as 1-5 percent of school age children in the United States (May et al., 2018). There is no cure for FASD, and elucidation of the underlying mechanisms which regulate development of these disorders is needed to generate effective therapies.

Fetal ethanol exposure can induce neuropathology in multiple brain regions, including the cerebellum. Human studies have demonstrated cerebellar susceptibility to ethanol exposure during development, including diminished cerebellar volume and white matter abnormalities, which likely contribute to impaired motor coordination, and learning and memory deficits often found in individuals with FASD (Riley and McGee, 2005; Lebel et al., 2008; Norman et al., 2009).

Rodent models of FASD have been valuable in studying fetal ethanol exposure. In mice for instance, the first two postnatal weeks coincide with the third trimester of human gestation (Clancy et al., 2001), a critical period for cerebellar development. During this time the cerebellum undergoes a period of secondary neurogenesis, cell migration, and synaptogenesis. This period also exhibits oligodendrocyte maturation and myelination (Rice and Barone, 2000; Camarillo and Miranda, 2007; Wilhelm and Guizzetti, 2016). Rodent studies using third trimester equivalent ethanol exposure paradigms have shown ethanol-induced cerebellar alterations associated with activation of both astrocytes and microglia. The activation states of these cells can affect their overall function, including changes in expression of pro-inflammatory molecules, suggesting ethanol-induced cerebellar neuroinflammation could be associated with FASD (Kane et al., 2011; Drew et al., 2015; Topper et al., 2015).

In recent years, RNA sequencing (RNAseq) has become more readily accessible and cost efficient making it a highly effective tool to assess ethanol-induced transcriptomic changes in the CNS (Farris and Mayfield, 2014; Berres et al., 2017; Erickson et al., 2019; Pinson et al., 2021). Using our neonatal model of FASD, in which mice were treated with ethanol on postnatal days (P) 4-9 and tissues harvested on P10, we recently reported that ethanol stimulated transcriptomic changes associated with cell cycle and microglia regulation, and oligodendrocyte lineages in the cerebellum (Pinson et al., 2021). In the current studies, we evaluated ethanol-induced transcriptomic changes in the cerebellum on postnatal days 5 and 6 in this FASD model. These findings may provide further insight into the underlying mechanisms associated with the early onset of FASD as well as identifying potential targets for clinical interventions and therapeutics.

## Materials and methods

### Animals

C57BL/6J mice were purchased from The Jackson Laboratory (Bar Harbor, ME; stock #000664) and housed in the federally approved Division of Laboratory Animal Medicine facility at the University of Arkansas for Medical Sciences (UAMS) where an in-house breeding colony was established to produce experimental animals. All animal use protocols were reviewed and approved by the UAMS Institutional Animal Care and Use Committee. Individually housed pregnant dams were kept on a 10:14 hour light:dark cycle in static cages on an open-air rack and were checked twice daily for birth of pups, with postnatal day 0 (P0) being designated as the day of birth. Cages were changed weekly or as needed. Dams were allowed unlimited access to food and water for the duration of the experiments. Experimental litters contained 4-8 neonates that were distributed among treatment groups, Ethanol (E) or vehicle Control (C) and were separated according to sex as evenly as possible for each individual litter. Handled-only, untreated Control animals were not included in this study, based on no difference for analogous endpoints from previous studies (Kane et al., 2011; Drew et al., 2015). On P4-5, Ethanol treated animals were administered 4 g/kg/day of ethanol in 20% intralipid (Fresenius Kabi, Uppsala, Sweden) while Control animals received 20% intralipid in which ethanol was substituted with an equal volume of water. Ethanol and water were administered via intragastric gavage. On P5 or P6, 24 hours after the last ethanol treatment on P4 or P5, respectively, animals were anesthetized using isoflurane vapor and transcardially perfused with phosphate-buffered saline containing 5 U/mL heparin. The brain was removed, the cerebellum was microdissected, flash-frozen in liquid nitrogen, and stored at −80°C until used for RNA isolation and subsequent sequencing. 1 male and 1 female Control, and 1 male and 1 female Ethanol were randomly selected from each of 3 litters for sequencing on P5 and P6 (N = 6 total litters, 3 male/3 female per treatment group, per timepoint). To determine mean Blood Ethanol Concentration (BEC), 3 separate litters were treated with ethanol as described above and blood was collected from half of each litter on either P4 or P5, 90 minutes after ethanol administration. Briefly, animals were anesthetized using isoflurane vapor and trunk blood was collected in heparinized capillary tubes following decapitation. Blood was centrifuged at 4000 RPM for 5 minutes and serum was removed for BEC determination using an Analox AM1 alcohol analyzer (Analox Technologies USA, Atlanta, GA) and companion Alcohol Reagent Kit (Analox #GMRD-113) according to manufacturer specifications. P4 mean BEC was 324.6 mg/dL ± 7.2 mg/dL SEM, (*n* = 7 male, 4 female) and P5 mean BEC was 333.7 mg/dL ± 12.6 mg/dL SEM, (*n* = 7 male, 5 female).

### Isolation of RNA, RNA-seq library preparation, and sequencing

Frozen cerebellar tissues were rapidly thawed and homogenized in Qiazol with 0.5 mm glass beads (Qiagen #13116-50) in a PowerLyzer 24 homogenizer (Qiagen #13155) for 30s at 3500 rpm. Total RNA was isolated using an miRNeasy Mini kit (Qiagen #217084) and DNA was removed with on-column DNase1 digestion (Qiagen #79254) following manufacturer protocol (Qiagen, Valencia, CA). RNA quantity was evaluated using the Qubit 3.0 fluorometer with the Qubit Broad-Range RNA Assay kit (Thermo Fisher Scientific, Waltham, MA). RNA quality was assessed using the Agilent Fragment Analyzer with the Standard Sensitivity RNA Gel Kit (Agilent Technologies, Santa Clara, CA). RNA-seq libraries were prepared using the Illumina TruSeq mRNA Library Prep Kit with TruSeq unique dual-indexed adapters (Illumina, San Diego, CA). Libraries were quantified with the Qubit 1X dsDNA High-Sensitivity NGS Gel Kit (Thermo Fisher), and additionally characterized for functionality with the KAPA Library Quantification Kit (Roche, Basel, Switzerland) and for fragment size using the Agilent Fragment Analyzer with the High-Sensitivity NGS Gel Kit (Agilent). According to manufacturer’s specification for clustering, library molarities were calculated followed by dilution and denaturation. Control and Ethanol-exposed animals were clustered on a high-output NextSeq 500 flow cell and paired-end sequenced with 150-cycle SBS kit for 2 × 75 reads (Illumina).

### Bioinformatic analysis

Raw RNA-sequence data [NCBI gene expression omnibus (GEO) series succession number GSE226532 (Edgar et al., 2002)] was analyzed to identify significant differences in mRNA gene expression and global biological pathways associated with alterations of cerebellar genes between Control and Ethanol treatment groups. Using the Nextflow RNAseq pipeline, nf-core/rnaseq (version 3.4) available at DOI: 10.5281/zenodo.1400710, RNAseq reads were quality-checked, trimmed, and aligned, with the resulting gene counts transformed to Log_2_ counts per million (CPM) and lowly expressed genes were filtered out (Liao et al., 2014). Libraries were normalized by trimmed mean of M-values (Robinson and Oshlack, 2010). To calculate differential gene expression, the Limma R package was used (Ritchie et al., 2015). Genes with an adjusted p-value (adj. *p* < 0.05) were considered statistically significant and Log_2_ fold change values were calculated for Ethanol compared to Control.

Heatmaps, principal component analysis (PCA), and volcano plots were generated in R from the processed differential gene expression data. Specifically, the *EnhancedVolcano* package was used to generate the volcano plots (Blighe et al., 2022). Utilizing the ‘‘Core Expression Analysis’’ in the QIAGEN Ingenuity Pathway Analysis (IPA) software (QIAGEN Inc.)^1^, pathway and network analysis were conducted. In IPA, the analysis parameter setting for “species” was set to “mouse” and the “tissues and cell lines” parameter was set to “brain”. The gene cut off criteria was set to an adj. *p* < 0.05. Once all analysis parameters were set, the analysis was run.

Publicly available single-cell RNA seq (scRNA-seq) resources were used to investigate the specific cell types and cellular processes that may be altered by ethanol exposure in the cerebellum in our dataset. We and others have used this analysis technique to deduce cell composition of bulk RNAseq tissue previously (Jew et al., 2020; Pinson et al., 2021). Subsequently, we compiled a list of 822 microglia associated genes (Supplementary Table 1A), 309 astrocyte associated genes (Supplementary Table 2), and 799 oligodendrocyte lineage associated genes (Supplementary Table 3) utilizing this approach (Zeisel et al., 2015; Artegiani et al., 2017; Sousa et al., 2018; Jurga et al., 2020; Ochocka and Kaminska, 2021).

Cell type specific gene lists were further characterized by transcripts associated with specific phenotypes for each of the glial cell populations. Microglia associated genes, for example, were subcategorized into transcripts related to homeostasis or neurodegenerative diseases (Keren-Shaul et al., 2017; Krasemann et al., 2017; Butovsky and Weiner, 2018; Tatsuyuki Matsudaira, 2022) (Supplementary Table 1B). Astrocyte associated genes were subcategorized into transcripts related to acute injury, chronic injury, or pan-injury, with pan-injury including genes associated with both acute and chronic reactive astrogliosis (Das et al., 2020) (Supplementary Table 2). Oligodendrocyte lineage cell associated genes were subcategorized into transcripts related to oligodendrocyte precursor cells (OPCs), committed oligodendrocyte precursor cells (COPs), newly-formed oligodendrocytes (NFOL), myelin forming oligodendrocytes (MFOL), and mature oligodendrocytes (MOL) (Zeisel et al., 2015, 2018; Artegiani et al., 2017) (Supplementary Table 3).

Each of these phenotype specific lists was cross-referenced to the transcripts significantly dysregulated by Ethanol (adj. *p* < 0.05) when compared to Control in our data set for both P5 and P6 and was tested for statistical significance. To accomplish this, R statistical software was utilized to generate individual z-scores for each transcript of interest and each experimental animal within a given phenotype list. These z-scores were then averaged across transcripts for each individual animal. Control versus Ethanol groups were then tested for statistically significant variance in R by two-tailed, student’s t-test, and graphical results were generated.

Our previous studies demonstrated that ethanol altered the expression of molecules associated with various stages of the cell cycle in our FASD model in which animals were treated with ethanol from P4-P9 and cerebellar RNA isolated on P10 (Pinson et al., 2021). In the current study, we performed similar analysis to determine if ethanol altered cell cycle progression during the onset of FASD in this model. Thus, from the Mouse Genome Database Gene Ontology Browser (Bult et al., 2019), we extracted gene lists associated with positive and negative regulation of both G1-S phase transition and G2-M phase transition. Average z-score analysis was conducted in a manner consistent with the glial cell types above.

## Results

### Cerebellar differential gene expression following the onset of ethanol exposure in the third trimester

**FIGURE 1:**
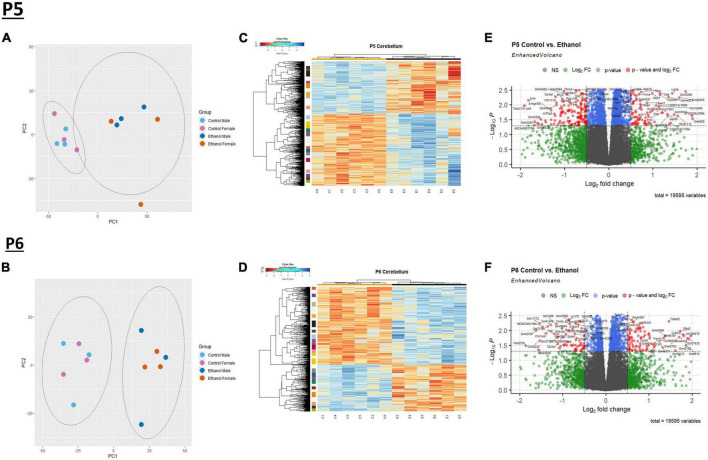
Ethanol-induced differential gene expression in the cerebellum. Principle component analysis (PCA) of the variance of genes in the cerebellum between ethanol (E) and control (C) at P5 **(A)** and P6 **(B)**. A heatmap and hierarchical clustering dendrogram of the of relative gene expression across samples for the significantly (adj. *p* < 0.05) altered genes for P5 **(C)** and P6 **(D)**. A volcano plot displaying fold change versus adjusted *p*-value of all detected genes in the cerebellum at P5 and P6. 2,440 of 19,595 total identified transcripts at P5 **(E)** and 1,348 of 19,595 total identified transcripts at P6 **(F)** displayed an adjusted *p* < 0.05 and Log_2_ fold change ≥ 0.5 or ≤ –0.5, shown in red. PCA, heatmaps, and volcano plots (*Enhanced Volcano* package) were generated using R statistical software. *n* = 3 males and 3 females per treatment group E or C.

We evaluated gene expression profiles at P5 and P6, 24 and 48 hours, respectively, after ethanol exposure began. First, a principal component analysis (PCA) was performed on male and female Control and Ethanol treated animals to evaluate distinct differences between the two datasets at P5 and P6 (Figures 1A, B). The first and second principal components encapsulate gene expression patterns that differentiate Control versus Ethanol treated animals. The PCA analysis suggested there were minimum sex differences in the ethanol regulation of gene expression, thus we combined sexes for the remainder of the analysis. Secondly, hierarchical clustering analysis using Pearson correlation was conducted on those genes that were identified as significantly dysregulated by ethanol (adj. *p* < 0.05) at P5 and P6 (Figures 1C, D). Volcano plot analysis identified 2,440 genes that were significantly dysregulated (adj. *p* < 0.05 and log_2_FC ± 0.5) at P5 and 1,348 genes at P6. Of the 2,440 genes at P5, 1,419 were upregulated and 1,021 were downregulated (Figure 1E). Of the 1,348 genes at P6, 840 were upregulated and 508 were downregulated (Figure 1F).

### Pathway and cellular function analysis of genes dysregulated by ethanol at P5 and P6

**FIGURE 2:**
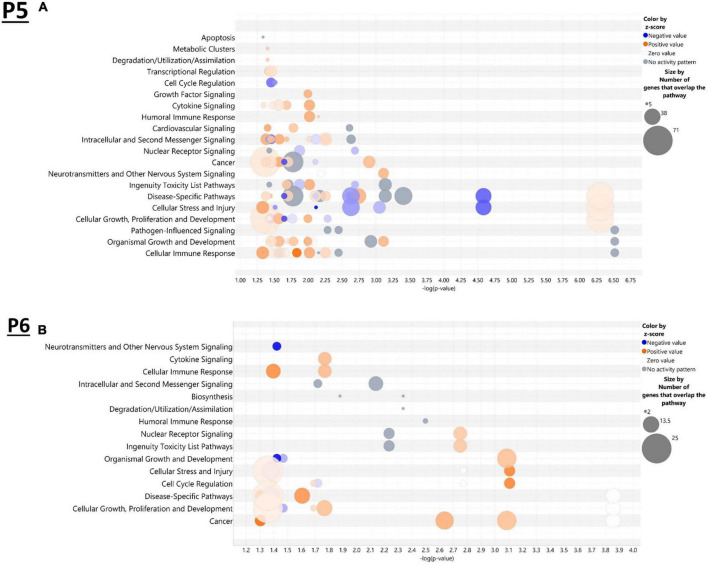
Top canonical pathways altered in the brain by ethanol exposure at P5 and P6. Qiagen Ingenuity Pathway Analysis (IPA) software was employed to assess the top canonical pathways altered by ethanol at P5 **(A)** and P6 **(B)**. All analyses were restricted to genes with an adj. *p* < 0.05 and analysis settings were set to “brain” in IPA. *n* = 3 males and 3 females per treatment group E or C.

IPA was utilized to determine specific pathways and cellular functions associated with genes significantly (adj. *p* < 0.05) dysregulated by ethanol. Results of the top canonical pathway categories altered by ethanol exposure common to P5 and P6, included neurotransmitters and other nervous system signaling, cytokine signaling, cellular immune response, intracellular and second messenger signaling, degradation/utilization/assimilation, humoral immune response, nuclear receptor signaling, ingenuity toxicity list pathways, organismal growth and development, cellular stress and injury, cell cycle regulation, disease-specific pathways, cellular growth and development, and cancer (Figures 2A, B). The top altered diseases and biological function categories of genes dysregulated by ethanol conserved between P5 and P6 included cell death and survival, neurological disease, organismal injury and abnormalities, cell-to-cell signaling and interaction, nervous system development and function, cellular growth and proliferation, tissue development, cellular assembly and organization, cellular movement, immune cell trafficking, cellular compromise, cellular function and maintenance, free radical scavenging, psychological disorders, organismal development, lipid metabolism, metabolic disease, molecular transport, small molecule biochemistry, cell morphology, developmental disorder, embryonic development, inflammatory response, and organ morphology (Tables 1 and 2). The tabular descriptions of the diseases and functions categories, including annotations, p-value, and associated transcripts that correlate with these diseases and biological function categories are listed in Supplementary Tables 4A for P5 and 4B for P6.

**TABLE 1:** P5-Top diseases and biological functions altered by ethanol exposure.

Category	*P*-value range
Cellular development	5.63E-05–4.91E-02
Nervous system development and function	5.63E-05–4.91E-02
Tissue development	5.63E-05–4.91E-02
Tissue morphology	7.94E-05–3.92E-02
Cellular growth and proliferation	7.32E-04–4.91E-02
Neurological disease	7.36E-04–4.87E-02
Organismal injury and abnormalities	7.36E-04–4.87E-02
Psychological disorders	7.36E-04–2.81E-02
Cell morphology	1.01E-03–3.85E-02
Cellular assembly and organization	1.01E-03–4.63E-02
Cellular function and maintenance	1.01E-03–4.63E-02
Organismal development	1.01E-03–4.86E-02
Cell death and survival	1.64E-03–3.34E-02
Embryonic development	1.64E-03–3.88E-02
Organ morphology	2.67E-03–3.81E-02
Behavior	4.39E-03–4.86E-02
Cancer	5.09E-03–4.87E-02
Free radical scavenging	7.05E-03–7.05E-03
Cardiovascular disease	7.5E-03–1.93E-02
Cell–mediated immune response	9.11E-03–1.93E-02
Cellular movement	9.11E-03–3.44E-02
Hematological system development and function	9.11E-03–3.88E-02
Immune cell trafficking	9.11E-03–2.23E-02
Lipid metabolism	9.11E-03–4.87E-02
Molecular transport	9.11E-03–4.87E-02
Small molecule biochemistry	9.11E-03–4.87E-02
Endocrine system disorders	9.57E-03–9.57E-03
Gastrointestinal disease	9.57E-03–9.57E-03
Metabolic disease	9.57E-03–3.17E-02
Organ development	1.62E-02–3.88E-02
Cardiovascular system development and function	1.93E-02–4.86E-02
Cell–to–cell signaling and interaction	1.93E-02–3.85E-02
Developmental disorder	1.93E-02–1.93E-02
Hereditary disorder	1.93E-02–1.93E-02
Inflammatory response	1.93E-02–1.93E-02
Organismal functions	1.93E-02–1.93E-02
Endocrine system development and function	2.15E-02–2.97E-02
Cellular compromise	2.23E-02–2.23E-02
Amino acid metabolism	3.85E-02–3.85E-02

All analyses were restricted to genes with an adj. *p* < 0.05 and analysis settings were set to “brain” in IPA. *n* = 3 males and 3 females per treatment group E or C.

**TABLE 2:** P6-Top diseases and biological functions altered by ethanol exposure.

Category	*P*-value range
Cell death and survival	4.7E-04–4.46E-02
Neurological disease	4.7E-04–4.93E-02
Organismal injury and abnormalities	4.7E-04–4.93E-02
Cardiovascular disease	1.18E-03–4.02E-02
Cell–to–cell signaling and interaction	1.18E-03–4.02E-02
Nervous system development and function	1.18E-03–4.97E-02
Cellular growth and proliferation	2.81E-03–4.97E-02
Tissue development	2.81E-03–4.97E-02
Cellular assembly and organization	4.61E-03–4.97E-02
Cardiovascular system development and function	4.73E-03–4.17E-02
Cellular movement	4.73E-03–2.61E-02
Hematological system development and function	4.73E-03–2.61E-02
Immune cell trafficking	4.73E-03–2.61E-02
Organ development	4.73E-03–4.73E-03
Tissue morphology	9.76E-03–4.97E-02
Cellular compromise	1.35E-02–2.52E-02
Cellular function and maintenance	1.35E-02–4.97E-02
Free radical scavenging	1.35E-02–1.35E-02
Psychological disorders	1.35E-02–4.93E-02
Organismal development	1.39E-02–4.97E-02
Carbohydrate metabolism	1.92E-02–1.92E-02
Lipid metabolism	1.92E-02–2.61E-02
Metabolic disease	1.92E-02–1.92E-02
Molecular transport	1.92E-02–2.61E-02
Small molecule biochemistry	1.92E-02–2.61E-02
Cell morphology	2.52E-02–4.97E-02
Cellular development	2.52E-02–4.97E-02
Developmental disorder	2.52E-02–2.52E-02
Embryonic development	2.52E-02–4.97E-02
Inflammatory response	2.52E-02–2.52E-02
Organ morphology	3.42E-02–4.02E-02

All analyses were restricted to genes with an adj. *p* < 0.05 and analysis settings were set to “brain” in IPA. *n* = 3 males and 3 females per treatment group E or C.

### Ethanol induced alterations in microglia phenotypic states at P5 and P6

**FIGURE 3:**
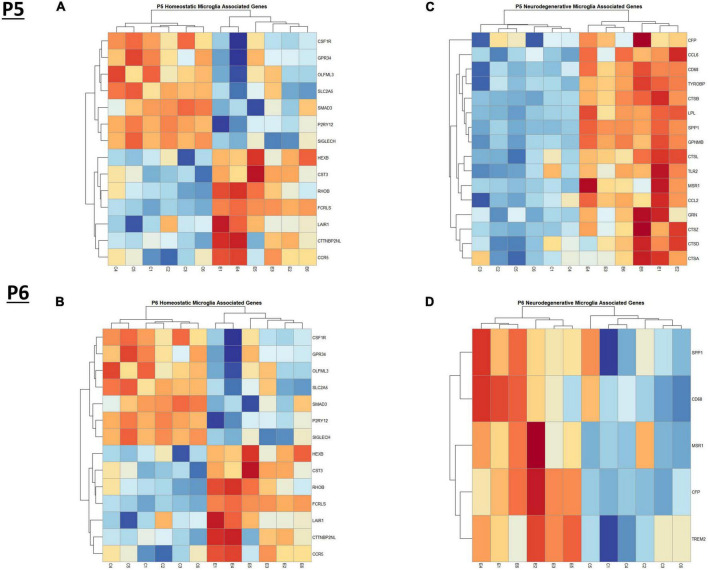
Heatmap and hierarchical clustering of microglia associated genes at P5 and P6. R statistical software was utilized to construct heatmaps and hierarchical clustering dendrogram of relative gene expression across samples for significantly altered (adj. *p* < 0.05) and categorized microglia associated genes as detailed in Methods. Microglia homeostatic associated gene expression is depicted in panel **(A)** for P5 and panel **(B)** for P6. Microglia neurodegenerative associated gene expression is depicted in panel **(C)** for P5 and panel **(D)** for P6.

**FIGURE 4:**
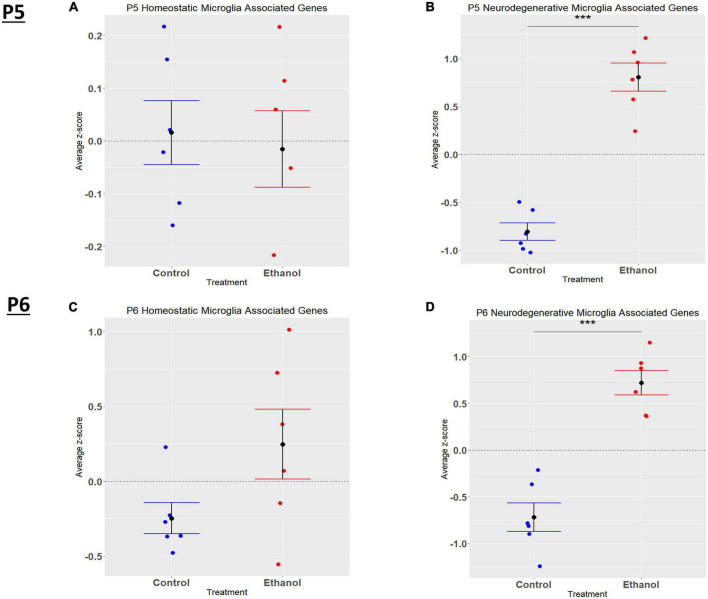
Microglia associated genes altered by ethanol exposure at P5 and P6 in the cerebellum. Microglia associated genes were extracted as detailed in Methods. R statistical software was utilized to generate individual z-scores for each transcript of interest and each experimental animal. These *z*-scores were then averaged across transcripts for each individual animal. Control versus Ethanol groups were then tested for statistically significant variance in R by two-tailed, student’s *t*-test, and graphical results were generated. Quantification by average *z*-score of homeostatic microglia associated genes at P5 **(A)** and P6 **(B)** and neurodegenerative microglia associated genes at P5 **(C)** and P6 **(D)**. *n* = 3 males and 3 females per treatment group E or C; ***p < 0.001.

In the current study, we compared the list of 822 microglia associated genes (described in the *Bioinformatic Analysis* subsection of the Materials and Methods) to the list of genes dysregulated by ethanol (adj. *p* < 0.05) at P5 and P6. We identified 175 microglia associated genes at P5 (Supplementary Table 5A) and 105 microglia associated genes at P6 (Supplementary Table 5B) that were significantly dysregulated by ethanol. We further categorized these 175 and 105 dysregulated microglia associated genes as being either typical of a homeostatic or a neurodegenerative phenotype (Supplementary Tables 5C, D), as previously defined (Supplementary Table 1B; Keren-Shaul et al., 2017; Krasemann et al., 2017; Butovsky and Weiner, 2018; Sousa et al., 2018; Tatsuyuki Matsudaira, 2022). Heatmaps illustrating relative gene expression across transcripts for significantly altered (*p* < 0.05) homeostatic and neurodegenerative disease microglial associated genes are depicted in Figures 3A, C for P5 and 3B and 3D for P6. A student’s t-test comparing the average z-scores across all relevant genes indicated that ethanol exposure did not significantly alter expression of homeostatic transcripts at P5, *p* = 0.7464 (Figure 4A) or P6, *p* = 0.0817 (Figure 4B), though P6 appears to approach significance. Examination of the average z-scores across all neurodegenerative disease associated microglia genes by student’s t-test showed ethanol induced a significant upregulation of these genes at both P5, *p* = 2.967e-06 (Figure 4C) and P6, *p* = 3.069e-05 (Figure 4D).

### Ethanol induced astrocyte phenotypic switch following neonatal ethanol exposure

**FIGURE 5:**
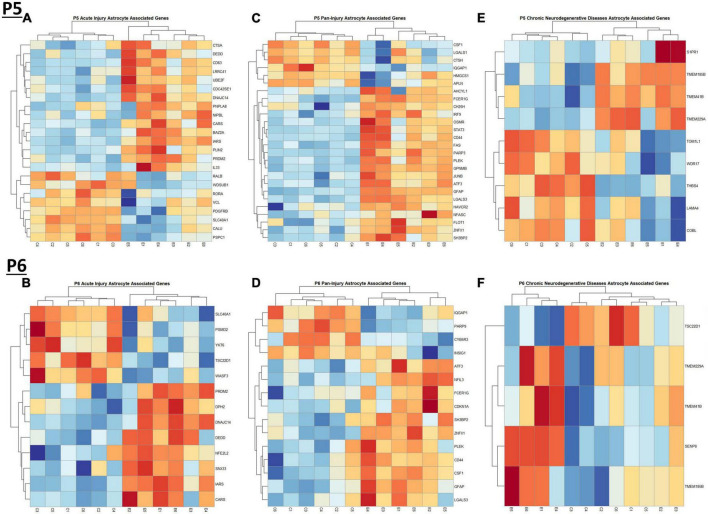
Heatmap and hierarchical clustering of astrocyte associated genes at P5 and P6. R statistical software was utilized to construct heatmaps and hierarchical clustering dendrogram of relative gene expression across samples for significantly altered (adj. *p* < 0.05) and categorized astrocyte associated genes as detailed in Methods. Astrocyte acute injury astrocyte associated gene expression is depicted in panel **(A)** for P5 and panel **(B)** for P6. Astrocyte pan-injury astrocyte associated gene expression is depicted in panel **(C)** for P5 and panel **(D)** for P6. Astrocyte chronic neurodegenerative diseases astrocyte gene expression is depicted in panel **(E)** for P5 and **(F)** for P6.

**FIGURE 6:**
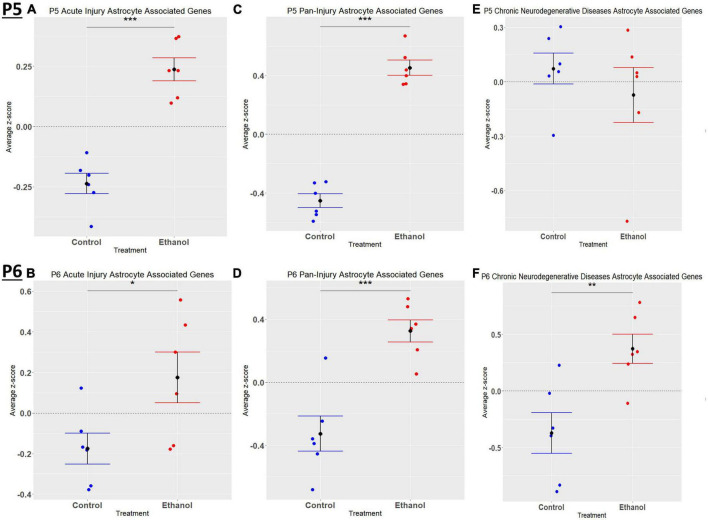
Astrocyte associated genes altered by ethanol exposure at P5 and P6 in the cerebellum. Astrocyte associated genes were extracted as detailed in Methods. R statistical software was utilized to generate individual *z*-scores for each transcript of interest and each experimental animal. These *z*-scores were then averaged across transcripts for each individual animal. Quantification by average *z*-score of acute injury astrocyte associated genes at P5 **(A)** and P6 **(B)**, pan-injury astrocyte associated genes at P5 **(C)** and P6 **(D)**, and chronic neurodegenerative diseases associated genes at P5 **(E)** and P6 **(F)**. *n* = 3 males and 3 females per treatment group E or C; *p < 0.05, **p < 0.01, ***p < 0.001.

When comparing the list of 309 astrocyte associated genes (described in the *Bioinformatic Analysis* subsection of the Materials and Methods) to our list of genes dysregulated by ethanol (adj. *p* < 0.05) at P5 and P6, we identified 58 astrocyte associated genes at P5 and 33 genes at P6. We further characterized these genes as belonging to an acute injury, chronic neurodegenerative diseases, or pan-injury phenotype (Supplementary Tables 6A, B; Das et al., 2020). Heatmaps illustrating relative gene expression across transcripts for significantly altered (*p* < 0.05) acute injury, pan-injury, and chronic neurodegenerative diseases associated genes are depicted in Figures 5A, C, E for P5 and Figures 5B, D, F for P6. A student’s *t*-test comparing the average z-scores of all relevant astrocyte associated genes indicated that ethanol induced a significant increase in astrocyte associated acute injury transcripts at both P5, *p* = 2.3e-05 (Figure 6A) and P6, *p* = 0.0376 (Figure 6B), and pan-injury transcripts at both P5, *p* = 1.465e-07 (Figure 6C) and P6, *p* = 0.0006 (Figure 6D). Astrocyte associated chronic neurodegenerative diseases transcripts showed no significant difference between Control and Ethanol groups for P5, *p* = 0.4234 (Figure 6E), but ethanol significantly increased expression of these transcripts at P6, *p* = 0.0073 (Figure 6F).

### Oligodendrocyte lineage cells have mixed effects under ethanol exposure at P5 and P6

**FIGURE 7:**
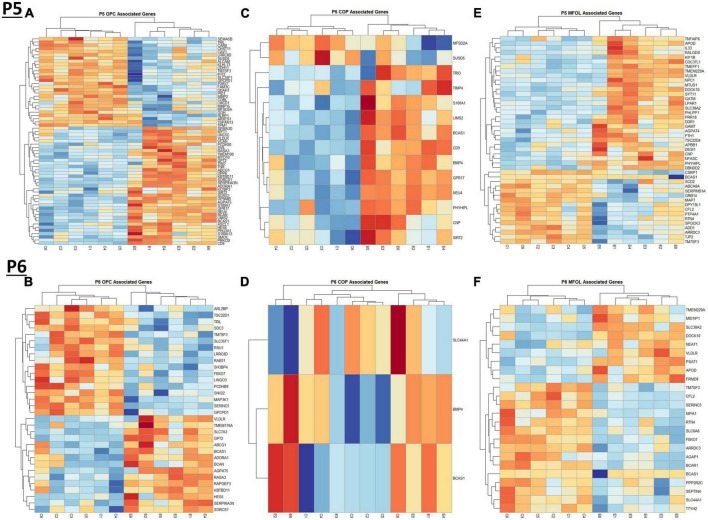
Heatmap and hierarchical clustering of oligodendrocyte lineage associated genes at P5 and P6. R statistical software was utilized to construct heatmaps and hierarchical clustering dendrogram of relative gene expression across samples for significantly altered (adj. *p* < 0.05) and categorized oligodendrocyte lineage associated genes as detailed in Methods. OPC associated gene expression is depicted in panel **(A)** for P5 and panel **(B)** for P6. COP associated gene expression is depicted in panel **(C)** for P5 and panel **(D)** for P6. MFOL associated gene expression is depicted in panel **(E)** for P5 and panel **(F)** for P6.

**FIGURE 8:**
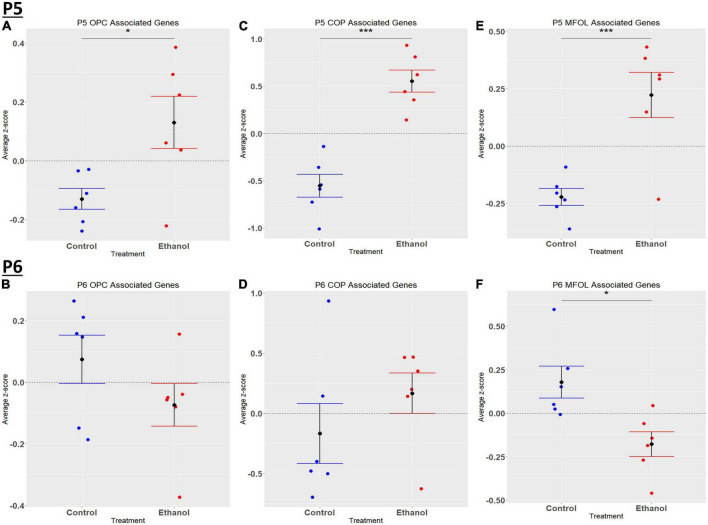
Oligodendrocyte lineage associated altered genes by ethanol exposure at P5 and P6 in the cerebellum. Oligodendrocyte lineage associated genes were extracted as detailed in Methods. R statistical software was utilized to generate individual *z*-scores for each transcript of interest and each experimental animal. These *z*-scores were then averaged across transcripts for each individual animal. Quantification by average z-score of OPC associated genes at P5 **(A)** and P6 **(B)**, COP associated genes at P5 **(C)** and P6 **(D)**, and MFOL associated genes at P5 **(E)** and P6 **(F)**. *n* = 3 males and 3 females per treatment group E or C; *p < 0.05, ***p < 0.001.

Comparing our list of genes dysregulated by ethanol (adj. *p* < 0.05) to the extracted list of oligodendrocyte lineage genes, at P5 we identified 65 OPCs, 14 COPs, 0 NFOL, 45 MFOL, and 1 MOL associated genes (Supplementary Table 7A), and at P6 we identified 32 OPCs, 3 COPs, 0 NFOLs, 24 MFOLs, and 0 MOL associated genes (Supplementary Table 7B). Heatmaps illustrating relative gene expression across transcripts for significantly altered (*p* < 0.05) OPC, COP, and MFOL associated genes are depicted in Figures 7A, C, E for P5 and Figures 7B, D, F for P6. Student’s *t*-test comparing the average z-scores across relevant genes showed that ethanol induced a significant upregulation of OPC associated transcripts at P5, *p* = 0.0219 (Figure 8A), with no significant effect on OPC associated transcripts at P6, *p* = 0.1887 (Figure 8B). In terms of COP associated genes, ethanol induced a significant upregulation at P5, *p* = 0.0219 (Figure 8C) but no effect at P6, *p* = 0.2924 (Figure 8D). Lastly, ethanol induced a significant upregulation of MFOL associated genes at P5, *p* = 0.0009 (Figure 8E), but a significant downregulation of MFOL associated genes at P6, *p* = 0.0340 (Figure 8F). NFOL and MOL were not analyzed.

### Alteration of cell cycle progression following ethanol exposure

**FIGURE 9:**
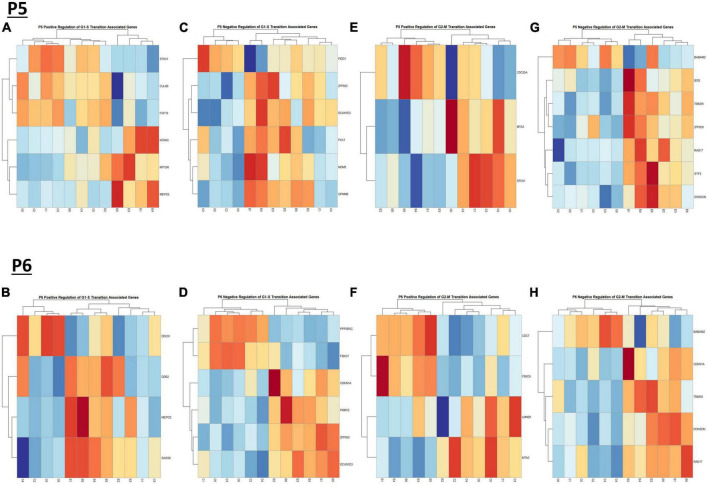
Heatmap and hierarchical clustering of cell cyle associated genes at P5 and P6. R statistical software was utilized to construct heatmaps and hierarchical clustering dendrogram of relative gene expression across samples for significantly altered (adj. *p* < 0.05) and categorized cell cycle associated genes as detailed in Methods. Positive regulation of G1-S transition associated gene expression is depicted in panel **(A)** for P5 and panel **(B)** for P6. Negative regulation of G1-S transition associated gene expression is depicted in panel **(C)** for P5 and **(D)** for P6. Positive regulation of G2-M transition associated gene expression is depicted in panel **(E)** for P5 and **(F)** for P6. Negative regulation of G2-M transition associated genes is depicted in panel **(G)** for P5 and panel **(H)** for P6.

**FIGURE 10:**
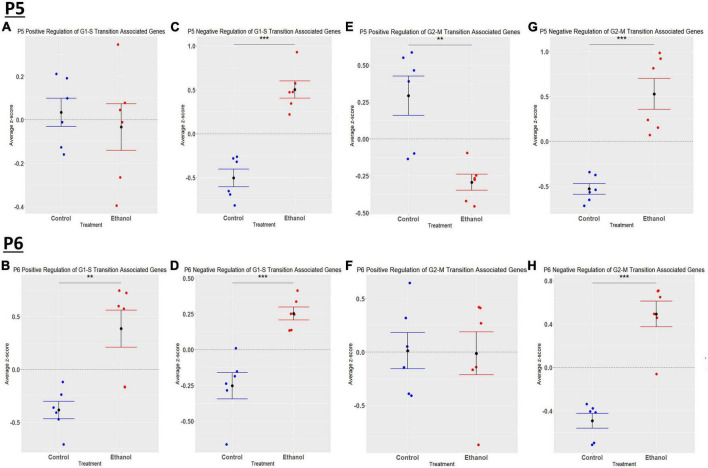
Ethanol-induced alterations in cell cycle regulation at P5 and P6 in the cerebellum. Gene list associated with positive and negative regulation of G1-S transition and positive and negative regulation of G2-M transition were extracted from the Mouse Genome Database. Genes associated with each cell cycle stage in our dataset at P5 and P6 were extracted as detailed in Methods. R statistical software was utilized to generate individual z-scores for each transcript of interest and each experimental animal. These z-scores were then averaged across transcripts for each individual animal. Quantification by average z-score of positive regulation of G1-S transition associated genes at P5 **(A)** and P6 **(B)**, negative regulation of G1-S transition associated genes at P5 **(C)** and P6 **(D)**, positive regulation of G2-M transition associated genes at P5 **(E)** and P6 **(F)**, and negative regulation of G2-M transition associated genes at P5 **(G)** and P6 **(H)**. *n* = 3 males and 3 females per treatment group E or C; **p < 0.01, ***p < 0.001.

We previously determined, using a third trimester human equivalent mouse model of FASD in which animals were treated with ethanol from P4-9 and cerebellar RNA isolated at P10 followed by RNASeq analysis, that ethanol increased the expression of molecules associated with the S and G2M phases of the cell cycle. In addition, our current IPA analysis (Figures 2A, B) suggests that cell cycle associated pathways may be altered by ethanol. Thus, we sought to determine if ethanol altered the expression of transcripts involved in cell cycle progression as early as P5 and P6. Using the Mouse Genome Database Gene Ontology Browser (Bult et al., 2019), genes associated with G1-S phase transition and G2-M phase transition cell cycle phases were extracted. Heatmaps illustrating relative gene expression across transcripts for significantly altered (*p* < 0.05) positive and negative regulation of G1-S transition associated genes and positive and negative regulation of G2-M transition associated genes are depicted in Figures 9A, C, E, G for P5 and Figures 9B, D, F, H for P6. For the positive regulation of G1-S phase transition, there was no significant difference between Control and Ethanol treated groups at P5, *p* = 0.6024 (Figure 10A); however, ethanol did induce a significant increase in those genes associated with this phase at P6, *p* = 0.0027 (Figure 10B). Ethanol induced a significant increase in those genes associated with the negative regulation of G1-S phase transition at both P5, *p* = 3.004e-05 (Figure 10C), and P6, *p* = 0.0006 (Figure 10D). Looking at the positive regulation of G2-M phase transition, ethanol induced a significant downregulation of genes associated with this phase at P5, *p* = 0.0021 (Figure 10E), with no significant changes between our Control and Ethanol groups at P6, *p* = 0.9278 (Figure 10F). Lastly, looking at the negative regulation of G2-M phase transition, ethanol induced a significant increase in those genes associated with this phase at both P5, *p* = 0.0002 (Figure 10G), and P6, *p* = 3.042e-05 (Figure 10H).

## Discussion

The current study was designed to evaluate the effects of ethanol on transcriptomic profiles in the cerebellum of early postnatal mice, which approximates the third trimester of gestation in humans. This is a period of secondary neurogenesis, and ethanol exposure at this time can result in FASD. The proposed studies were also designed to evaluate the effects of ethanol at early times following exposure to begin to assess transcriptomic changes that may contribute to initial development of FASD. Pathway analysis suggested that ethanol may alter immune related pathways at P5 and P6 soon after initial ethanol exposure. We previously demonstrated that ethanol induced microglial activation, production of pro-inflammatory cytokines and chemokines, as well as neuron cell loss in animals treated with ethanol from P4-P9 and tissue harvested at P10 (Kane et al., 2011, 2021; Drew et al., 2015; Pinson et al., 2021). Others have used similar FASD models and have also observed ethanol induced neuroinflammation (Topper et al., 2015; Zhang et al., 2018). Thus, the current studies suggest that ethanol-induced neuroinflammation occurs rapidly following ethanol exposure and may contribute to the initial neuropathology associated with FASD. In an attempt to define possible mechanisms by which ethanol induces early immune activation in the current studies, we identified immune related transcripts whose expression was strongly altered by ethanol in our transcriptomic data. At P5 and P6, top upregulated transcripts (Supplementary Tables 5A, B) included SPP1, CCL3, C5AR1, C3AR1, MSR1, and CD14. At P5, but not P6, CCL2 transcript levels were highly increased by ethanol. SPP1, which is also termed osteopontin, is a secretory molecule expressed by a variety of immune cells which has functions including immunomodulation, chemotaxis, and cell adhesion (Lin et al., 2022). SPP1 plays a role in alcohol liver disease (Apte et al., 2005; Seth et al., 2006; Lebel et al., 2008; Das et al., 2022). In the presence of ethanol, SPP1 binds to integrins and CD44 and activates transcription factors AP-1 and NF-κB (Das et al., 2005; Bellahcène et al., 2008). These transcription factors regulate the expression of pro-inflammatory cytokines that amplify the immune response and are also linked to addictive behaviors (Crews et al., 2017). AP1 consists as a dimer of Fos and Jun proteins, and it is interesting the ethanol increased the expression of Fos and Jun transcripts at P5 (Supplementary Table 5A). Furthermore, CCL2 and CCL3, target genes of NF-κB, are induced by ethanol and are key mediators of CNS inflammation and alcohol drinking behavior (Blednov et al., 2005; He and Crews, 2008). We previously demonstrated that ethanol induced the expression of CCL2 in animal models of FASD (Drew et al., 2015) as well as adult models of alcohol use disorder (Kane et al., 2014). Complement receptor C3AR1 expression is induced by ethanol resulting in altered phagocytosis by microglia (Kalinin et al., 2018). Previous studies also indicated that C5AR1 is involved in alcohol-induced inflammation (Blednov et al., 2005; He and Crews, 2008). Collectively, these results suggest potential mechanisms by which ethanol-induced neuroinflammation may contribute to the early onset of neuropathology associated with FASD.

Microglia play a role in several developmental homeostatic functions, including synapse development, plasticity, and maintaining the health of neurons, which are altered in FASD (Drew and Kane, 2014). Microglia are the primary resident immune cell in the CNS and become activated in response to a variety of stimuli (Lynch et al., 2010). Ethanol exposure has previously been demonstrated to result in microglial activation and production of pro-inflammatory cytokines and chemokines that may contribute to the neuropathology associated with FASD (Kane et al., 2011; Drew et al., 2015). Activated microglia are responsible for aiding in immune functions including phagocytosis, antigen presentation, and generation of inflammatory cytokines and chemokines (Ransohoff and Perry, 2009; Saijo and Glass, 2011; Ransohoff and Brown, 2012). Our IPA analysis indicated that exposure to ethanol resulted in microgliosis of the brain at both P5 and P6. Microgliosis occurs during pathogenic insults to the CNS (Li and Zhang, 2016). Traditionally, microglia activation has been separated into either an M1 pro-inflammatory phenotype or an M2 anti-inflammatory phenotype (Franco and Fernández-Suárez, 2015; Tang and Le, 2016). However, recent literature suggests that microglia phenotypes do not fit into this simple binary system (Franco and Fernández-Suárez, 2015). Because microglia phenotypes are complex, several microglial phenotypes corresponding to different diseases and physiological states have been described. However, two microglial gene expression profiles appear across multiple studies - homeostatic and neurodegenerative disease associated. Homeostatic microglia are believed to aid in synaptic plasticity and synaptogenesis, neurogenesis, and immune cell recruitment (Butovsky and Weiner, 2018). The neurodegenerative disease related microglia phenotype results from insult to the CNS, and microglia lose their homeostatic signature and gain a chronic inflammatory signature (Holtman et al., 2015; Moore et al., 2015; Paolicelli et al., 2022). Although there are a variety of neurodegenerative diseases, assessment of microglia phenotype during these disease states have identified a common neurodegenerative disease related microglia phenotype (Naj et al., 2014; Moore et al., 2015; Keren-Shaul et al., 2017; Butovsky and Weiner, 2018). When examining microglia phenotypic states in the current study, ethanol induced a phenotypic switch at both P5 and P6 in the cerebellum, resulting in upregulation of neurodegenerative disease associated transcripts. The expression of homeostatic associated genes was not altered at P5 or P6 in the current studies. However, three of the top four most strongly ethanol-downregulated microglial associated molecules at P5 are considered homeostatic molecules (Supplementary Tables 1B, 5A). Additionally, microglia homeostatic associated molecules at P6 trended toward significance. This might suggest ethanol decreases the expression of at least a subset of homeostatic microglial associated transcripts in the current study which could in turn change the phenotype of microglia and alter specific developmental functions.

Astrocytes, like microglia, play a role in immune responses in the CNS and produce cytokines and chemokines, nitric oxide, and reactive oxygen species (Ransohoff and Brown, 2012). Astrocytes also play roles in maintaining the blood brain barrier and neurotransmitter levels, along with regulating energy balance and modulating synaptic plasticity (Santello et al., 2019). They also have a significant immune function, mediating both pro-inflammatory and anti-inflammatory activities in response to CNS insult (Dong and Benveniste, 2001). Astrocyte production of immune mediators is suspected to contribute to neuropathology associated with FASD (Guizzetti et al., 2014; Wilhelm and Guizzetti, 2016). Astrocytes may become reactive in response to various stimuli, resulting in astrogliosis/astrocytosis. During astrogliosis/astrocytosis, astrocytes undergo a phenotypic change which has historically been referred to result in an A1 reactive phenotype characterized as being neurotoxic or an A2 reactive phenotype described as being neuroprotective (Zamanian et al., 2012; Liddelow et al., 2017). However, classifying reactive astrocytes into these two categories does not appear to be adequate. A recent meta-analysis of mouse transcriptomic studies aimed to better categorize astrocyte reactive states (Das et al., 2020). The nomenclature used in this study classified reactive astrocytes as having three different phenotypes; acute injury, chronic neurodegenerative diseases, or pan-injury which has characteristics of both acute injury and chronic neurodegenerative diseases phenotypes (Das et al., 2020). In the current study, at P5, our IPA analysis indicated that ethanol treatment resulted in alterations related to the development of astrocytes, formation of astrocyte precursor cells, and quantity of astrocytes, and at P6, our IPA analysis revealed that ethanol treatment stimulated astrocytosis and gliosis of astrocytes in the cerebellum. These results suggest that 24 h following initial ethanol exposure, astrocyte development and quantity is altered, and after 48 h of ethanol exposure, those astrocytes that are present are becoming reactive. We also revealed that at both P5 and P6, ethanol stimulated an acute injury and pan-injury reactive astrocyte phenotype, with a chronic neurodegenerative diseases phenotype also being seen at P6, but not P5. LPS is known to induce an immune response in the CNS, and was demonstrated to trigger an acute injury astrocyte phenotype (Das et al., 2020). Like LPS, ethanol is believed to trigger immune responses, at least in part, through activation of TLR4 signaling pathways (Blanco et al., 2005; Floreani et al., 2010; Alfonso-Loeches et al., 2014). Therefore, in this model of FASD, it is possible that ethanol stimulates an acute or pan-injury reactive phenotypic state at both P5 and P6, possibly through activation of TLR4. It will be important in the future to define the mechanisms by which ethanol alters astrocyte phenotype and immune responses and how this may contribute to ethanol-induced neuropathology associated with FASD.

Oligodendrocytes generate the myelin sheath which wraps axons to promote the conduction of electrical impulses (Baumann and Pham-Dinh, 2001). Prior to myelination, oligodendrocytes undergo a series of differentiation steps, beginning as OPCs and terminating as mature myelinating oligodendrocytes (Bradl and Lassmann, 2010; El Waly et al., 2014). Studies of children and adolescents with FASD have demonstrated white matter abnormalities, suggesting that ethanol has a long-lasting impact on myelination (Wilhelm and Guizzetti, 2016). During development, OPCs migrate from their origin to their functional site where they differentiate into mature myelinating oligodendrocytes. In rodents, myelin formation occurs abundantly during the first two postnatal weeks; however, OPC differentiation into mature myelinating oligodendrocytes can occur throughout life (El Waly et al., 2014). Ethanol effects on myelination in animal models of FASD have begun to be investigated. Studies have demonstrated that postnatal ethanol exposure in rats resulted in myelin deficits and aberrant eye-blink conditioning, which is a cerebellum-dependent learning task (Rufer et al., 2012). Using a similar postnatal model of FASD, ethanol was demonstrated to decrease both proliferating OPCs and mature oligodendrocytes in the corpus callosum. Interestingly, the effects of ethanol on OPCs depended on the ontogenetic origin of these cells. Furthermore, although OPC and oligodendrocyte numbers recovered by adulthood, the myelin microstructure remained aberrant as determined by diffusion tensor imaging (Newville et al., 2017). Myelin was also aberrant in third trimester models of FASD in sheep (Dalitz et al., 2008) and oligodendrocyte apoptosis was abundant in a third trimester FASD model in macaques (Creeley et al., 2013). We have recently demonstrated that ethanol dysregulated transcripts associated with OPCs, pre-myelinating oligodendrocytes, and mature oligodendrocytes in a postnatal mouse model of FASD in which animals were treated with ethanol from P4-9 and cerebellum isolated at P10 (Niedzwiedz-Massey et al., 2021a). Using the same model, we demonstrated that ethanol decreased the expression of transcripts associated with OPCs and mature oligodendrocytes in the hippocampus (Niedzwiedz-Massey et al., 2021b). In the current study, we evaluated the effects of ethanol on immature oligodendrocyte lineage cells and mature myelinating oligodendrocytes at P5 and P6. Interestingly, at P5, ethanol induced a significant increase in transcripts associated with both immature oligodendrocyte lineage cells and myelinating oligodendrocytes. This increase in the expression of oligodendrocyte related transcripts, particularly those associated with OPCs may result from an initial compensatory response to the toxic effects of ethanol. OPCs are highly proliferative during this stage of development and continue to proliferate until a balanced number of OPCs is reached (Hughes et al., 2013). If this balance is disrupted, perhaps as a response to ethanol, OPCs are triggered to continue proliferation in order to maintain a consistent pool (Hughes et al., 2013). At P6, ethanol did not alter the expression of transcripts associated with early-stage oligodendrocytes but decreased the expression of transcripts associated with mature oligodendrocytes. The decrease in myelin forming oligodendrocyte transcripts seen at P6 could result in decreased myelination observed in FASD. These results suggest that after two days of consecutive ethanol exposure one begins to see a depletion in myelinating oligodendrocytes.

Developmental alcohol exposure is known to affect cell cycle regulation and apoptosis related events (Anthony et al., 2008). At P5, we saw no significant effect of ethanol on transcripts associated with the positive regulation of G1-S transition, and an increase in transcripts involved in negative regulation of this cell cycle phase. Ethanol also decreased the expression of positive regulators and increased the expression of negative regulators of the G2-M phase at P5. Collectively, these data suggest that ethanol negatively impacts G1-S and G2-M transitions at P5. At P6, ethanol increased the expression of transcripts involved in both positive and negative regulation of the G1-S transition. Ethanol did not alter the expression of positive regulators but increased the expression of negative regulators of the G2-M transition at P6. Collectively, these data show no clear effect of ethanol on G1-S transition and a decrease in G2-M transition at P6. During early postnatal development in rodents, cells in the external germinal layer of the cerebellum undergo vast proliferation to generate a substantial pool of cerebellar granule progenitors, which eventually form cerebellar neurons (Miale and Sidman, 1961; Altman, 1997; Li et al., 2002). Additionally, cerebellar interneurons are born and migrate to their final destination to form synaptic connections with Purkinje cells (Schilling et al., 2008; Leto and Rossi, 2012). Our data suggest that early third trimester equivalent ethanol exposure halts cell cycle progression in both the synthesis and mitosis phases, preventing cells from replicating. The accumulation of cells in these phases could result in a potential decrease in the pool of cerebellar granule cells that will eventually form mature neurons while also limiting the generation of interneurons. Having deficits in both granule cells and interneurons of the cerebellum could contribute to the reduced cerebellar volume and aberrant motor function and cognitive deficits seen in individuals with FASD. However, further studies are needed to evaluate this possibility.

The current study demonstrated that ethanol altered the transcriptomic profile in the cerebellum in a postnatal model of FASD. However, there are several limitations in the experimental design which should be considered when interpreting these data. For example, it should be acknowledged that alterations in transcript expression in the current RNASeq analysis will need to be confirmed by RT-PCR analysis. Furthermore, these transcript changes need to be evaluated at the level of protein expression. Increasing the sample size would also add confidence that the observed results will be experimentally reproducible. The levels of alcohol used in the current studies are also relatively high. Future studies are needed to determine transcriptomic changes in mice treated with more moderate levels of ethanol. It also should be acknowledged that some of the transcriptomic changes observed may not result solely or specifically due to ethanol but could result from a more general acute stress response to high doses of ethanol. Future studies are also needed to determine which transcripts and pathways altered by ethanol in these studies may contribute to the pathogenesis of FASD and thus represent potential targets for FASD therapy. We also note that the expression of more transcripts was observed at P5 after a single dose of ethanol than at P6 after two doses of ethanol. Additional studies are needed to determine the potential relevance of these temporal changes in transcriptomic profiles to FASD.

In conclusion, the current studies demonstrate that ethanol has profound effects on the transcriptomic profile in the developing cerebellum, early following initial ethanol exposure which may be critical in the development of FASD. IPA analysis indicated that ethanol likely alters pathways involved in immune signaling and cell cycle. With regard to glia, ethanol induced an increase in transcripts related to a neurodegenerative microglia phenotype along with an increase in transcripts associated with acute and pan-injury reactive astrocyte phenotypes at both P5 and P6 and additionally a chronic neurodegenerative disease astrocyte phenotype at P6 but not P5. Lastly, ethanol induced differing effects in the expression of genes associated with immature oligodendrocyte lineage cells and myelinating oligodendrocytes. These studies may begin to unravel the effects of ethanol during the onset of FASD.

## Data availability statement

The data discussed in this publication have been deposited in NCBI’s Gene Expression Omnibus and are accessible through GEO Series accession number: GSE226532 (https://www.ncbi.nlm.nih.gov/geo/query/acc.cgi?acc=GSE226532).

## Ethics statement

This animal study was reviewed and approved by University of Arkansas for Medical Sciences, Institutional Animal Care and Use Committee (IACUC).

## Author contributions

PD, CK, and AM: conceptualization. KH, JD, and PD: writing—original draft. KH, JD, TR, AM, CK, and PD: writing—review and editing. KH, JD, and TR: investigation and visualization. KH and JD: formal analysis. PD and AM: supervision. All authors had access to the data for the study, made substantial contributions to the manuscript, approved the submitted version of the manuscript, and take responsibility for the accuracy and integrity of the data.

## Footnotes

1
https://digitalinsights.qiagen.com/IPA
